# Regulation of Inflammasomes by Application of Omega-3 Polyunsaturated Fatty Acids in a Spinal Cord Injury Model

**DOI:** 10.3390/cells10113147

**Published:** 2021-11-12

**Authors:** Maryam Baazm, Victoria Behrens, Cordian Beyer, Omid Nikoubashman, Adib Zendedel

**Affiliations:** 1 Department of Anatomy, School of Medicine, Arak University of Medical Sciences, Arak 3819693345, Iran; dr.baazm@arakmu.ac.ir; 2 Institute of Neuroanatomy, Uniklinik RWTH Aachen, 52074 Aachen, Germany; vbehrens@ukaachen.de (V.B.); cbeyer@ukaachen.de (C.B.); 3 Department of Neuroradiology, University Hospital RWTH, 52074 Aachen, Germany; onikoubashma@ukaachen.de

**Keywords:** omega-3 fatty acids, spinal cord injury, inflammasome

## Abstract

Omega-3 polyunsaturated fatty acids (PUFA n3) ameliorate inflammation in different diseases and potentially improve neurological function after neuronal injury. Following spinal cord injury (SCI), inflammatory events result in caspase-1 mediated activation of interleukin-1 beta (IL-1b) and 18. We aim to evaluate the neuroprotective potency of PUFA n3 in suppressing the formation and activation of inflammasomes following SCI. Male Wistar rats were divided into four groups: control, SCI, SCI+PUFA n3, and SCI+Lipofundin MCT (medium-chain triglyceride; vehicle). PUFA n3 or vehicle was intravenously administered immediately after SCI and every 24 h for the next three days. We analyzed the expression of NLRP3, NLRP1, ASC, caspase-1, IL-1b, and 18 in the spinal cord. The distribution of microglia, oligodendrocytes, and astrocytes was assessed by immunohistochemistry analysis. Behavioral testing showed significantly improved locomotor recovery in PUFA n3-treated animals and the SCI-induced upregulation of inflammasome components was reduced. Histopathological evaluation confirmed the suppression of microgliosis, increased numbers of oligodendrocytes, and the prevention of demyelination by PUFA n3. Our data support the neuroprotective role of PUFA n3 by targeting the NLRP3 inflammasome. These findings provide evidence that PUFA n3 has therapeutic effects which potentially attenuate neuronal damage in SCI and possibly also in other neuronal injuries.

## 1. Introduction

Spinal cord injury (SCI) is associated with several temporary or permanent pathological changes resulting in loss of physiological nervous function [1]. The pathological changes of SCI involve primary and secondary damage. The primary injury results from the initial insult and is characterized by destruction of neural tissue and blood vessels, whereas the secondary phase starts after a few minutes and lasts for weeks post the mechanical injury and is identified with an extensive neurological injury such as oxidative stress, ischemia, and inflammation [2].

Neuroinflammation is one of the principal factors in the pathogenesis of SCI which is mainly mediated by the activation of immune cells (such as macrophages/microglia) [3]. Shortly after SCI, immune cells are activated and subsequently secrete a series of pro-inflammatory cytokines to recruit blood-borne leukocytes to the injured site. Activation of cytoplasmic inflammasome complexes is regarded as the essential step of neuroinflammation and a key trigger for neuronal death called pyroptosis. Inflammasome complexes are involved in the activation of caspase-1 which catalyzes the cleavage of pro-interleukins (including interleukin-18 [IL-18] and IL-1β) into their active forms [4]. Most inflammasomes consist of a sensor protein: NOD-like receptor (NLR), an adapter molecule: apoptosis-associated speck-like protein containing a Caspase-recruitment domain (ASC, also known as Pycard), and an enzymatic component: Caspase 1 (CASP1). It has been shown that activation of inflammasomes such as NLR Family Pyrin Domain Containing 3 (NLRP3) is involved in pathogenesis of SCI [2]. Considering the variety of proteins involved in the regulation of inflammasome formation, it can be suggested that therapies targeting inflammasome components might be useful to inhibit inflammasome activation and their destructive effects in damaged tissue [5,6].

It has been reported that an alteration in lipid plasma membrane occurs following SCI [7]. There is some evidence that indicates the correlation between dyslipidemia and the degree of neurological problems in SCI patients [8,9]. It has been shown that the degree of neurological involvement in SCI subjects, whether considered as motor grade or neurological level, is associated with decreased levels of total cholesterol and high-density lipoprotein cholesterol (HDL-c). In addition, SCI patients have lower serum levels of total cholesterol, triglycerides, HDL-c, and low-density lipoprotein cholesterol (LDL-c) than the general population [10,11]. Omega-3 polyunsaturated fatty acid (PUFA n3), including eicosapentaenoic acid (EPA), docosahexaenoic acid (DHA), and other family members, have potential roles in CNS development and deficiency in these components increases the rate of depression during adulthood [10]. Considering the essential roles of this product in improving brain function during some neurological diseases, it has to be taken into account that PUFA n3 cannot be synthesized inside the body and needs to be taken up with food [11]. There is growing evidence that the administration of PUFA n3 reveals anti-inflammatory and neuroprotective properties on the brain and exhibits an improvement in neurobehavioral function following traumatic brain injury and stroke [12,13]. PUFA n3 has been shown to stimulate macrophages to inhibit NLRP3 complex activity and reduce pro-inflammatory cytokine production, including IL-1b, IL-18, and IL-6 after traumatic brain injury [14,15]. In addition, treatment of obese people or adipocytes with fish oil results in suppression of NLRP3 expression [16]. In the context of SCI, it has been shown that a preventive PUFA n3-rich diet results in a significantly improved functional outcome in a rat SCI model [17]. Furthermore, the presence of higher PUFA n3 levels seems to result in a decrease in the expression of pro-inflammatory cytokines post SCI [18]. However, a possible reduction in inflammasome activation through PUFA n3 was not verified in this context.

According to the above conceptual studies, it seems that the use of PUFA n3 is a promising approach to reduce inflammation in some inflammatory disorders and traumatic injuries, including SCI. Given that the anti-inflammatory mechanisms of PUFA n3 in SCI are poorly understood, in this study, we aimed to investigate whether omega-3 polyunsaturated fatty acids exert their anti-inflammatory activity via inhibition of inflammasome activation in our SCI experimental setting.

## 2. Materials and Methods

All experimental and animal care protocols were approved by the Review Board for the Care of Animal Subjects of the district government (Tehran, Iran; code: IR.NIMAD.REC.1396.123). Animals were fed a standard chow and water ad libitum and were exposed to a 12 h light/dark cycle, at a temperature of 24 ± 1 °C.

### 2.1. Animals

Adult male Wistar rats (200–250 gr, Pasteur, Iran) were divided randomly into four groups (*n* = 8): control, SCI, SCI+ Lipo MCT (vehicle), SCI+ PUFA n3. PUFA n3 was administered at a dose of 250 nmol/kg, in a volume of 5 mL/kg. The intravenous injection was performed in five min and through a permanent catheter located in the external jugular vein immediately after injury and every 24 h for 3 d. Lipofundin MCT (Lipo MCT, B. Braun, Melsungen, Germany) was used as a vehicle which contains PUFA n3 without omega-3 fatty acid substitution. Two blood samples were taken from each animal: before oil emulsion administration and before sacrificing. Physiological NaCl in the same volume was used to replace the drawn blood.

### 2.2. Surgical Procedure

In this experimental study, animals were deeply anesthetized with 2.0% isoflurane in O2 (Abbott, Ludwigshafen, Germany) and positioned in a stereotactic apparatus. During surgery, a warm plate was used to avoid local hypothermia. After shaving the surgical areas, skin incision and blunt dissection of the muscle layers over the area of the vertebral T10 level (spinal T8/9) were performed. SCI was performed according to our previous studies [19,20]. Briefly, using two adjustable forceps, the spine processes of both T7 and T13 were fixed. After laminectomy at the T10 vertebra, the spinal cord was compressed by placing a 50 g weight on the exposed dura mater. The tip of the weight had an area of 11.0 mm^2^ with a concave shape that ensured equal distribution of the pressure on the spinal cord. Subsequently, for the insertion of a permanent catheter in the external jugular vein, a small incision was performed in midline neck skin. After exposing the external jugular vein, a catheter was inserted, passed subcutaneously, exteriorized, and sutured to the skin. After the operation, muscles and skin layers were sutured and animals received saline rehydration (2 mL) and Baytril (0.3 mL, subcutaneously, twice a day) to prevent infection. To avoid bladder infections, the bladders of the animals were manually voided twice a day. Thirty minutes after surgery, animals received an intrajugular injection of vehicle (Lipo MCT) or PUFA n3 (Sigma-Aldrich, Darmstadt, Germany). Then, 72 h or 7 d after injury, the animals were sacrificed under deep anesthesia and immediately transcardially perfused with 150–200 mL phosphate-buffered saline (PBS; Sigma-Aldrich, Darmstadt, Germany). The tissue corresponding to the epicenter of the injured spinal cord was removed and processed for further studies.

### 2.3. Behavioral Testing

Hindlimb locomotor function of the animals was evaluated using an open field locomotor scale, described by Basso, Beattie, and Bresnahan (BBB) from complete paralysis (score 0) to normal locomotion (score 21), to assess recovery after contusion injuries once a day for 7 d post SCI [21]. The spinal cord functions were assessed according to the hind limb movements, body weight support, forelimb to hind limb coordination, and whole-body movements. The evaluation was performed by an investigator blinded to the treatment.

### 2.4. Fatty Acid Analysis in Blood Samples

For measuring fatty acids in plasma samples, the HPLC (high-performance liquid chromatography) method and mass spectrometry were used. For isolating plasma from whole blood, at first, blood samples were kept at room temperature for 30 min, then centrifuged for 10 min at 6000 rpm. The upper phase which contained plasma was used for analyzing fatty acids. 500 μL methanol was added to each sample. The solution was incubated with 300 μL NaOH (10 M) at 80 °C for 1 h and then cooled at room temperature for 10 min. The neutralization was followed by adding 300 μL of 58% acetic acid. The sample was diluted with methanol and by adding 10 μL internal standard solution (ISTD) and was prepared for measurement. The fatty acid profile was determined with LC-MS/MS analysis using an Agilent 6460 Triplequad mass spectrometer with a Jetstream ion source operating in negative mode (Agilent Technologies, Santa Clara, CA, USA) coupled with Agilent 1200 HPLC (degasser, binary pump, well plate sampler, thermostated column compartment). As the stationary phase, a Phenomenex Kinetex Column (150 mm × 2 m, 2.6 μm; Phenomenex, Aschaffenburg, Germany) was used.

The chromatography was performed with acetonitrile and 0.1% formic acid as mobile phase under gradient conditions. It started at 30% and decreased to 2% within 10 min. The flow rate was 0.4 mL/min during the 16.5 min run time. The injection volume was 5 μL. The drying gas was adjusted at 210 °C/7 L/min, nebulizer at 45 psi, sheath gas at 350 °C/11 L/min. The capillary voltage was optimized at 4000 V (negative) and the nozzle voltage at 1500 V (negative). The sample concentrations were determined using linear calibration curves based on the relative peak area dependent on the target-compound/ISTD concentration ratios.

### 2.5. Luxol Fast Blue Staining

For determining myelinated nerve fiber, Luxol fast blue (LFB, Sigma, MI, USA) staining was performed. The spinal cord was fixed with 4% formaldehyde (Sigma, Taufkirchen, Germany), dehydrated, embedded in paraffin, and sectioned (5 μm). After deparaffinization and rehydration, the sections were incubated with LFB solution (0.01%) overnight at 56 °C. After rinsing the excess stain and differentiating the slides in lithium carbonate, the myelinated nerves were stained in blue whereas the demyelinated regions appeared in white. Stained samples were observed with a light microscope (Olympus CX310, Tokio, Japan) and the images were captured with a digital camera (Olympus, Tokio, Japan). A total of 10 sections per animal were considered for analyzing LFB stained regions (myelinated regions). For quantification of myelinated regions, blue color intensity was measured using Image J software. We calculated the percentage of myelinated regions in the white matter of SC by dividing the LFB-stained area by the total selected area multiplied by 100.

### 2.6. Immunohistochemistry (IHC)

To perform IHC staining, the paraffin slices was deparaffinized, rehydrated, and unmasked by Tris/EDTA pH 9.0 buffer and subjected for immunohistochemistry using the Vectastain-DAB Kit (Vector Laboratories, Burlingame, CA, USA). After antigen retrieval, sections were blocked by incubation with 10% goat serum (Sigma, Taufkirchen, Germany) for 30 min. The sections were incubated with primary antibodies against IBA-1, GFAP, and OLIG2 overnight at 4 °C. After that, the appropriate secondary antibodies were added to each section for 2 h at room temperature. Diaminobenzidine (DAB) was used as chromogen substrate. Finally, sections were counterstained, dehydrated, and mounted. For quantification, the positive cells were counted using the ImageJ software. Four slices of each animal were analyzed.

### 2.7. RNA Extraction and Real-Time PCR

After sampling, the tissue from the epicenter of the injury was used to evaluate gene expression. At first, total RNA was extracted using peqGold RNATriFast (PeqLab, Erlangen, Germany) according to the manufacture’s protocol. The purity of the extracted RNA was determined using 260/280 ratios of optical density from each sample (Nanodrop 1000, PeqLap, Erlangen, Germany). 1 μg of total RNA was used for complementary DNA synthesizing using the MMLV reverse transcription (RT)-kit and random hexanucleotide primers (Invitrogen, Carlsbad, CA, USA). The expression of IL-1b, IL-18, ASC, NLRP1, and NLRP3 genes as well as cyclophilin A (CycloA; as housekeeping gene) in all experimental groups was studied by quantitative reverse transcriptase-polymerase chain reaction (qRT-PCR). The reaction system of qRT-PCR was a mixture consisting of 2 μL reverse-transcribed cDNA, 2 μL RNAse-free water (Invitrogen, Carlsbad, CA, USA), 5 μL 2× SensiMix SYBR and Fluorescein (Bioline, Memphis, TN, USA), and 0.5 μL of the respective primers (10 pmol/μL). Primer sequences are listed in Table 1. Each sample was loaded in duplicate and qRT-PCR was performed in the MyIQ detection system (Biorad, München, Germany). The expression ratio was calculated by the ΔΔCt-method using qbase+ software (Biogazelle, Zwijnaarde, Belgium).

### 2.8. Enzyme-Linked Immunosorbent Assay

Concentrations of both mature IL-1b (900-K91, Peprotech, Hamburg, Germany) and IL-18 (ABIN416245, antibodies-online GmbH, Aachen, Germany) were measured with enzyme-linked immunosorbent assay (ELISA) kits following the manufacturer’s protocol. Briefly, epicenter of the spinal cord was homogenized in PBS (0.02 mol/L, pH 7.0–7.2) and centrifuged at 5000× *g* for 10 min at 4 °C. Then, the supernatant was collected and used for analysis by ELISA. Samples were loaded in duplicates and assayed at absorbance rates of 405 nm for IL-1b, and 450 nm for IL-18, respectively. Concentrations were calculated from standard curves and are given as picograms per milligram of the entire protein.

### 2.9. SDS PAGE and Western Blot

NLRP3, ASC, IL-1b, and caspase-1 protein were analyzed using Western blotting. At first, the spinal cord tissue was lysed and suspended in ice-cold RIPA buffer (50 mM Tris–HCl [pH 8], 1% Nonidet P40, 0.1% sodium dodecyl sulfate [SDS], 0.5% sodium deoxycholate and protease inhibitor cocktail). Then, the BCA Protein Assay Kit (Pierce, Hägersten, Sweden) was used for determining protein concentration according to the manufacturer’s instructions. Next, the same amounts of each protein sample (30 μg per lane) were loaded, separated by 8–12% (*v*/*v*) discontinuous SDS-polyacrylamide gel electrophoresis (SDS-PAGE), and transferred onto a polyvinylidene fluoride (PVDF) membrane (Roche, Basel, Switzerland). Then, blocking was performed with 5% skimmed milk in Tris-buffered saline containing 0.05% Tween 20 (TBS-T) for 1 h at room temperature. The PVDF membranes were incubated overnight with diluted primary antibodies at 4 °C (Table 2). The membranes were washed with TBS-T and incubated with a peroxidase-conjugated goat anti-rabbit (Bio-Rad, Berkeley, CA, USA) secondary antibody for 2 h at room temperature. Finally, the bands were detected using the enhanced chemiluminescence method (ECL plus, Pierce Scientific, Waltham, MA, USA) according to the manufacturer’s protocol. The intensities of the specific bands were normalized to glyceraldehyde-3-phosphate dehydrogenase (GAPDH) in the same blot using ImageJ software (Free Java software provided by the National Institute of Health, Bethesda, MD, USA).

### 2.10. Data Analysis

All data are given as means± standard error (SEM). GraphPad Prism 8 (GraphPad Software Inc., San Diego, CA, USA) was used for statistical analyses. The Brown–Forsythe test was performed to test for equal variances and normal distribution was tested with the Shapiro–Wilk test. If necessary, data were transformed via Box-Cox-Y to achieve homoscedasticity. Statistical differences between experimental groups were analyzed by one-way analysis of variance (ANOVA) followed by Dunnett post hoc test or two-way ANOVA (for BBB test) followed by Tukey post hoc test was used for parametric data. Non-parametric data were analyzed with the Kruskal–Wallis test followed by Dunn’s multiple comparisons or Friedman test. *p*-value ≤ 0.05 was considered statistically significant.

## 3. Results

### 3.1. Plasma Levels of DHA, EPA and ALA

**Figure 1:**
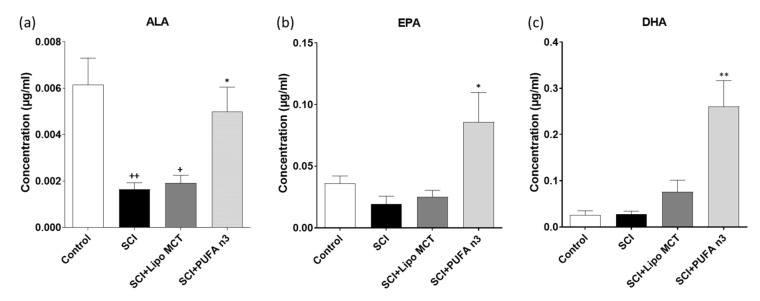
Fatty acid concentration in plasma. Blood plasma analysis of ALA (**a**), EPA, (**b**) and DHA (**c**) from the different experimental groups by HPLC 72 h after SCI. Data represent the means ± SEM from eight animals per group. Note the increase in amount of plasma fatty acids after PUFA n3 treatment. + *p* ≤ 0.05 and ++ *p* ≤ 0.01: SCI vs. Control; * *p* ≤ 0.05 and ** *p* ≤ 0.01: SCI + PUFA n3 vs. SCI + Lipo MCT.

In order to determine the systemic levels of ALA, EPA, and DHA, we examined the blood plasma of animals that received injections of PUFA n3 or vehicle (Lipo MCT) emulsions and compared them to basal levels obtained from control animals. Our data showed that plasma levels of ALA were significantly reduced in the SCI group and after PUFA n3 treatment returned back to basal level (Figure 1a). EPA and DHA were significantly increased after PUFA n3 treatment (Figure 1b,c).

### 3.2. Effects of PUFA n3 on Inflammatory Cytokines and Inflammasome Components

**Figure 2:**
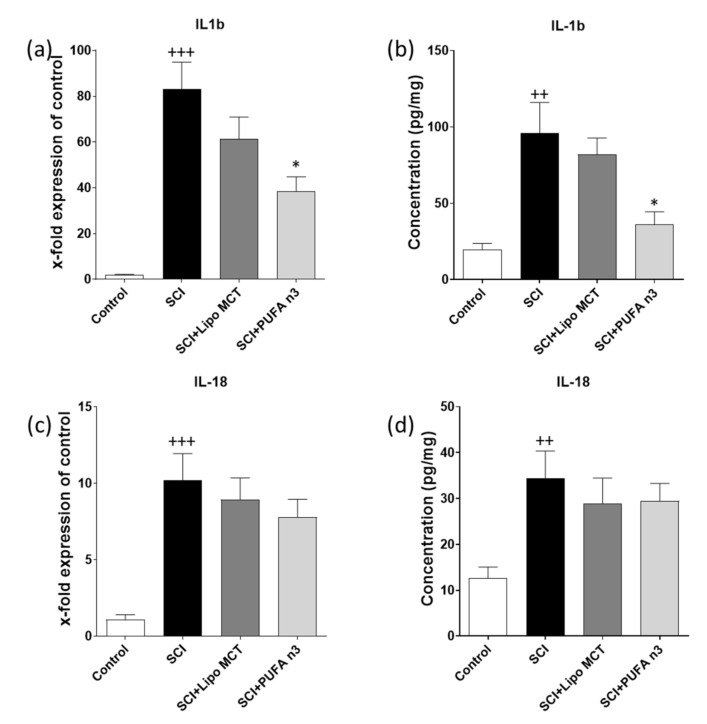
Cytokine expression after SCI in the lesion region. Analysis of IL-1b (**a**) and IL-18 (**c**) gene expression in the epicenter of spinal cord 72 h after SCI. Measurement of the respective protein amounts of the inflammatory cytokines, IL-1b (**b**) and IL-18 (**d**), in the spinal cord after SCI using ELISA. Note that SCI significantly increased the levels of inflammatory cytokines and PUFA n3 decreased the SCI-induced IL-1b production. ++ *p* ≤ 0.01 and +++ *p* ≤ 0.001: SCI vs. Control; * *p* ≤ 0.05: SCI+PUFA n3 vs. SCI+Lipo MCT.

**Figure 3:**
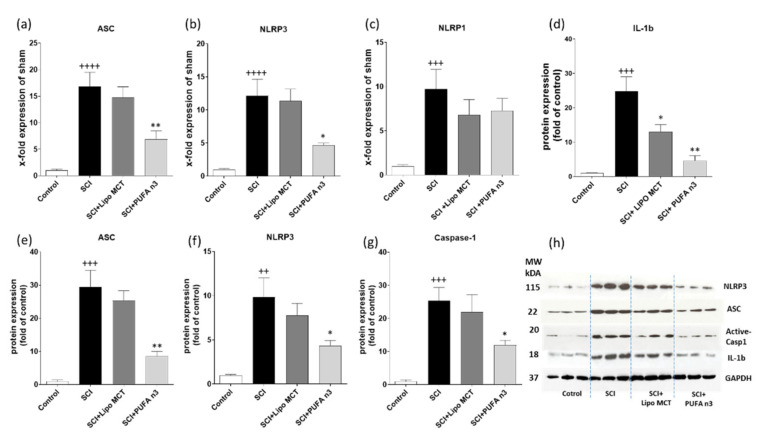
Inflammasomes after SCI in the lesion region. Expression pattern of inflammasome components ASC (**a**), NLRP3 (**b**) and NLRP1 (**c**) in different experimental groups 72 h post SCI was analyzed by real-time PCR. Western blot analysis of the inflammasome components IL-1b, ASC, NLRP3 and caspase-1 (**d**–**h**). ++ *p* ≤ 0.01, +++ *p* ≤ 0.001 and ++++ *p* ≤ 0.0001: SCI vs. Control; * *p* ≤ 0.05, and ** *p* ≤ 0.01: SCI+PUFA n3 vs. SCI+Lipo MCT.

To determine whether PUFA n3 substitution affects the SCI-induced inflammatory reaction following SCI, we measured the protein and gene levels of IL-1b and 18 by ELISA and qRT-PCR respectively (Figure 2). According to our previous timeline study, inflammasome components are up-regulated with a peak at 72 h post SCI, therefore, the effect of PUFA n3 on inflammasomes was determined at this time point (Figure 3) [6]. As expected, the mRNA levels and protein concentrations of these two cytokines were significantly up-regulated following SCI. Treatment with PUFA n3 could only decrease IL-1b mRNA and protein concentration (Figure 2a,b) but did not affect IL-18 expression (Figure 2c,d).

In the next step, to determine if this anti-inflammatory effect of PUFA n3 is mediated through inflammasome regulation, we measured the inflammasome complex. Therefore, we analyzed the mRNA and protein levels of various inflammasome components (Figure 3). Our results showed that SCI induces the expression of NLRP3, ASC, and caspase-1 at both mRNA and protein levels (Figure 3a,b,d–h) and PUFA n3 could significantly attenuate these effects (Figure 3a,b,d–h). Interestingly, PUFA n3 inhibits the protein expression of mature IL-1b post injury (Figure 3d). The elevated NLRP1 mRNA expression was not affected by PUFA n3 substitution (Figure 3c).

### 3.3. Effects of PUFA n3 on Remyelination

**Figure 4:**
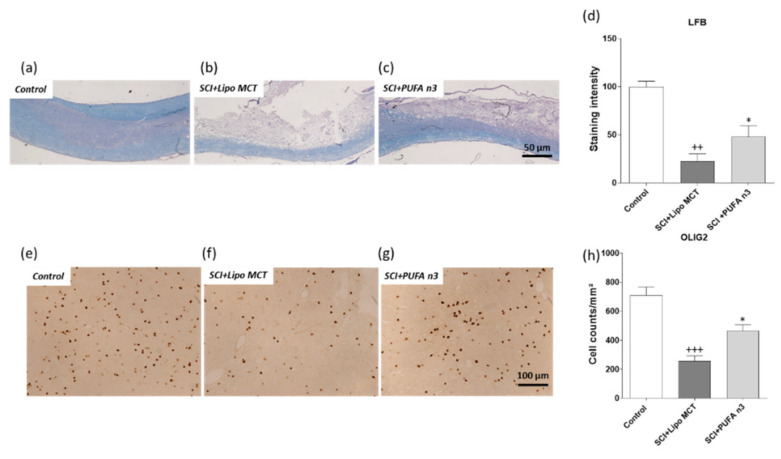
Demyelination and number of oligodendrocytes in the spinal cord after injury. LFB staining of SC tissue (lesion region) from the experimental groups control, SCI+Lipo MCT, and SCI+PUFA n3 7 d after injury (**a**–**d**). Myelin staining intensity (LFB) was markedly reduced in SC post SCI. PUFA n3 application protected SC from demyelination post SCI in epicenter. Representative microphotographs of OLIG2-positive cells (pan-oligodendrocyte marker) of the different experimental groups given above (**e**–**g**). Quantification of OLIG2-positive cells/mm^2^ (**h**). The number of oligodendrocytes is reduced in both groups post SCI but with significantly higher numbers of remaining OLIG2-positive cells in PUFA n3 treated rats. ++ *p* ≤ 0.01 and +++ *p* ≤ 0.001: SCI+ Lipo MCT vs. Control; * *p* ≤ 0.05: SCI+PUFA n3 vs. SCI+Lipo MCT.

To demonstrate the effect of PUFA n3 on the prevention of demyelination, sagittal sections of SC were stained with LFB. In control operated animals, LFB staining of myelin in the SC was mainly restricted to the white matter (Figure 4a). The microscopic observations revealed that at 7 d post SCI, LFB staining was strongly reduced with almost no staining visible in the dorsal column in the epicenter (Figure 4b,d). In addition, most of the ventral column also showed a partial reduction in staining. In sharp contrast, the loss of myelin was significantly prevented in PUFA n3 treated animals (Figure 4c,d). To further examine whether the beneficial effect of PUFA n3 on demyelination is correlated with improved survival of oligodendrocytes in treated animals, we conducted immunohistochemical detection with an anti-OLIG2 (oligodendrocytes) antibody. The histological quantifications of oligodendrocytes showed that SCI induced a significant loss of OLIG2-positive cells in the SC (Figure 4f,h). In animals treated with PUFA n3, we found higher numbers of OLIG2-positive cells compared to control rats after SCI (Figure 4g,h).

### 3.4. Effects of PUFA n3 on Glial Cells and Functional Recovery

**Figure 5:**
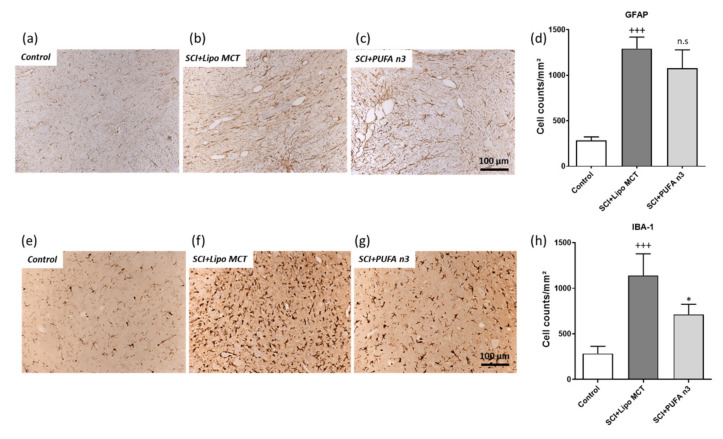
Astrocytes and microglia in the spinal cord after injury. Assessment of number of astrocytes 7 d post SCI using GFAP (**a**–**d**). SCI induces a significant increase in GFAP-positive cells 7 d after SCI. Treatment with PUFA n3 did not affect the SCI-induced astrogliosis. (**e**–**g**) Show the quantitative evaluation of microglia/macrophages (IBA-1) cell numbers in SC 7 d after SCI and representative microphotographs of the different experimental groups. Note that microglia/macrophage responses are significantly reduced in treated animals. +++ *p* ≤ 0.001: SCI vs. Control; * *p* ≤ 0.05: SCI+PUFA n3 vs. SCI+Lipo MCT. Scale bar: 100 μm.

Since gliosis is one of the main characteristic features post SCI, our next approach was to investigate the effect of PUFA n3 on astrocytes and microglia populations. Our immunohistochemical staining revealed that SCI induces astro- (GFAP) and microgliosis (IBA-1) at 7 days post SCI (Figure 5b,d,f,h). PUFA n3 substitution significantly suppressed the microgliosis (Figure 5g,h) but did not affect the number of GFAP-positive cells post SCI (Figure 5c,d).

**Figure 6:**
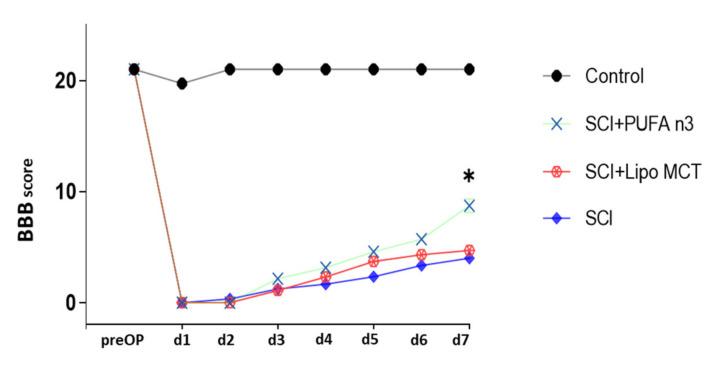
Functional impairment after SCI. BBB locomotion test was used to assess the motor function in hind-limbs during the first 7 d after SCI. There was no significant difference from d 1 to 6 between vehicle and treated groups. On d 7, we observed a significantly improved functional recovery in PUFA n3 treated animals. * *p* ≤ 0.05: SCI+PUFA n3 vs. SCI+Lipo MCT.

Finally, to investigate the effect of PUFA n3 on locomotor recovery after SCI, we performed BBB scoring for 7 consecutive days post injury. During the first 6 days after insult, there was no significant difference between treated and control animals (Figure 6). At day 7 post injury, the PUFA n3 group showed a significantly higher BBB scoring compared to the SCI group which indicates that PUFA substitution led to improved recovery of locomotor abilities in SCI animals.

## 4. Discussion

PUFA n3, a polyunsaturated fatty acid present in seafood, is an important component of the cell membrane, which is needed to maintain the membranes’ flexibility [22]. Recent evidence shows that PUFA n3 can modulate several processes that contribute to the secondary damage in the CNS. For example, PUFA n3 has antioxidant and anti-inflammatory effects in different diseases [23,24]. Several possible mechanisms may underlie the anti-inflammatory role of PUFA n3: it inhibits chemotaxis, reduces expression of adhesion molecules such as intercellular adhesion molecule (ICAM)-1 and vascular cell adhesion molecule (VCAM)-1, and decreases inflammatory chemokine production [25]. There is evidence that PUFA n3 plays a role in suppressing the inflammatory responses in diseases such as rheumatoid arthritis, inflammatory bowel disease, and cardiovascular disease [26]. Further, it has been demonstrated that the use of PUFA n3 fish oil for 6 weeks by obese subjects declines circulatory inflammatory cytokines [16].

In an experimental SCI rat model, PUFA n3 reduces apoptosis and improves neuronal survival and locomotor outcomes when injected via the tail vein for 7 d. In addition, animals fed with a PUFA n3-enriched diet for 8 weeks before SCI, exhibited lower motor and sensory dysfunction [17]. In contrast, it has been reported that treatment with omega 6 worsens the outcome after SCI in adult rats [27]. Since neuroinflammation is one of the main hallmarks of SCI, understanding the possible underlying mechanisms of the anti-inflammatory role of PUFA n3 is very critical. In this study, we administered PUFA n3 through the jugular vein immediately after SCI and every 24 h for the next three days after injury induction and evaluated the NLRP3 inflammasome activity. The increase in the plasma concentration of ALA, EPA, and DHA 72 h post injury confirms the effectiveness of the chosen method. The reason to choose this route of application was based on our previous study in which different routes of applications were tested [28]. Our results showed that PUFA n3 suppressed the SCI-induced expression of the NLRP3 inflammasome complex, at both mRNA and protein levels. PUFA n3 suppressed ASC and also caspase-1 activation.

The inflammatory cytokine production was shown to be induced as early as 15 min following contusion of the rat spinal cord. Caspase-1 which converts immature IL-1b and IL-18 into their mature forms is rapidly activated after injury and the levels of IL-1b rise during the first hours after injury [2,6]. Therefore, the therapeutic interventions for minimizing the destructive effects of SCI should take place in the early phase of SCI. In this study, we administered PUFA n3 immediately and every 24 h for the next three days after the operation to induce its neuroprotective and anti-inflammatory effects. There are few studies that describe the effect of omega-3 on inflammasome regulation in other animal models [15,29]. It was demonstrated that PUFA n3 suppresses inflammation by reducing NLRP3 inflammasome activation in activated macrophages [30]. Furthermore, this agent has the ability to reduce atherosclerosis progression by activating autophagy in macrophages and inhibiting the NLRP3 inflammasome activity [31]. In a type 2 diabetic mouse model, omega-3 fatty acid application decreased inflammasome activity and leads to the prevention of insulin resistance induced by high food diet [14]. In addition, Lin et al. (2017) have shown that pre-treatment of animals with omega-3 fatty acids inhibits the secretion of IL-1b and IL-18 through inactivation of NLRP3 inflammasome in a traumatic brain injury model [15]. They describe that omega-3 inhibits the NLRP3 complex through G protein-coupled receptor 40 (GPR40) mediated pathway [15]. GPR40 is one of the PUFA receptors which is expressed in CNS and involved in neurogenesis and neuronal function and β-Arrestin-2 (ARRB2) which is located downstream of GPR40 directly interacts with NLRP3 and inhibits inflammatory responses [15,32]. PUFA n3 could suppress inflammatory cytokines, ameliorate stroke, and limit neuronal damage when administered immediately and 12 h after transient middle cerebral artery occlusion [13]. According to these results, it seems that the inflammasome complex is an essential target for PUFA n3 to exert its anti-inflammatory activity in some diseases.

The NLRP3 inflammasome is present in different cell types (such as microglia and astrocytes) of the spinal cord which activates caspase-1 after injury [20,33,34,35]. Microglia are one of the regulators of neuroinflammation in different diseases [36,37]. We showed that PUFA n3 also reduces the SCI-induced microgliosis, suggesting that PUFA n3 treatment could inhibit the activation or recruitment of immune cells to the injury site and therefore leads to attenuation of secreted inflammatory cytokines [38]. Since microglia are one of the main sources of inflammasomes in the spinal cord it might be assumed that PUFA n3 treatment could alleviate the inflammasome activation via suppression of microgliosis in our experimental model of SCI [33,34,35].

We observed that PUFA n3 protects oligodendrocytes post SCI. This cell type is known to play an important role in maintaining myelin integrity and regulating axon growth in the spinal cord [39,40]. After SCI, they undergo both necrosis and apoptosis acutely, which causes demyelination and impairs axon function and survival [41]. Therefore, the protection of oligodendrocytes and consequently myelin production has beneficial effects on the functional outcomes post SCI [42]. In parallel to the preservation of oligodendrocytes, we found that PUFA n3 application results in improved functional recovery and also reduces the SCI-induced demyelination which leads to better BBB score outcomes.

In this study, we are able to demonstrate that PUFA n3 provides solid neuroprotection in the spinal cord by protecting oligodendrocytes and myelin which is paralleled by a reduction in behavioral deficits. In addition, PUFA n3 treatment alleviates the local NLRP3 inflammasome activation within the spinal cord and reduces neuroinflammation and microgliosis. We assume that the modulation of neuroinflammatory processes at the inflammasome platform, besides other well-described neuroprotective pathways activated by PUFA n3, represents an important mechanism to transmit neuroprotection after acute spinal injuries.

## Figures and Tables

**Table 1:** List of primer sequences used for real-time PCR.

Genes		Primer Sequences (5′ to 3′)
CycloA	F	GGCAAATGCTGGACCAAACAC
	R	TTAGAGTTGTCCACAGTCGGAGATG
IL-1b	F	TGGCAACTGTCCCTGAACTC
	R	GTCGAGATGCTGCTGTGAGA
IL-18	F	GGACTGGCTGTGACCCTATC
	R	TGTCCTGGCACACGTTTCTG
NLRP1b	F	GGGGCAGCCAAATCAAGTTC
	R	TGAGCGGTCATTGCAACTCT
NLRP3	F	TCTGTTCATTGGCTGCGGAT
	R	GCCTTTTTCGAACTTGCCGT
ASC	F	GCTGCAGATGGACCCCATAG
	R	ACATTGTGAGCTCCAAGCCA

**Table 2:** List of antibodies used in this study.

Antibody	Company	Western Blot	IHC
ASC	Santa Cruz, Dallas, TX, USA	1:1000	-
OLIG2	Santa Cruz, Dallas, TX, USA	-	1:1000
IBA-1	Abcam plc, Cambridge, UK	-	1:2500
CASPASE-1	Santa Cruz, Dallas, TX, USA	1:1000	-
NLRP3	Bioss, Woburn, MA, USA	1:1000	-
GFAP	Abcam plc, Cambridge, UK	-	1:1000
GAPDH	Sigma Aldrich, Saint Louis, MI, USA	1:4000	-

## Data Availability

The primary data can be requested by mail from the corresponding author.
